# Comparing Intensive Trauma-Focused Treatment Outcome on PTSD Symptom Severity in Older and Younger Adults

**DOI:** 10.3390/jcm10061246

**Published:** 2021-03-17

**Authors:** Ellen M. J. Gielkens, Ad de Jongh, Sjacko Sobczak, Gina Rossi, Agnes van Minnen, Eline M. Voorendonk, Linda Rozendaal, Sebastiaan P. J. van Alphen

**Affiliations:** 1 Clinical Center of Excellence for Older Adults with Personality Disorders, Mondriaan, 6419 PJ Heerlen-Maastricht, The Netherlands; s.sobczak@mondriaan.eu (S.S.); b.van.alphen@mondriaan.eu (S.P.J.v.A.); 2 Personality and Psychopathology Research Group (PEPS), Vrije Universiteit Brussel (VUB), 1050 Brussels, Belgium; Gina.Rossie@vub.be; 3 Psychotrauma Expertise Centre (PSYTREC), 3723 MB Bilthoven, The Netherlands; a.d.jongh@acta.nl (A.d.J.); vanminnen@psytrec.com (A.v.M.); voorendonk@psytrec.com (E.M.V.); rozendael@psytrec.com (L.R.); 4 Academic Centre for Dentistry Amsterdam (ACTA), University of Amsterdam and VU University Amsterdam, 1081 LA Amsterdam, The Netherlands; 5 School of Health Sciences, Salford University, Manchester M6 6PU, UK; 6 Institute of Health and Society, University of Worcester, Worcester WR2 6AJ, UK; 7 School of Psychology, Queen’s University, 18-30 Malone Road, Belfast BT9 5 BN, UK; 8 Department of Psychiatry and Neuropsychology, School for Mental Health and Neuroscience (MHeNs), Maastricht University, 6226 NB Maastricht, The Netherlands; 9 Behavioural Science Institute (BSI), Radboud University Nijmegen, 6525 HR Nijmegen, The Netherlands; 10 Department of Medical and Clinical Psychology, Tilburg University, 5037 AB Tilburg, The Netherlands

**Keywords:** post-traumatic stress disorder, intensive treatment, older adults, trauma-focused treatment, prolonged exposure, EMDR therapy

## Abstract

Objective: To examine the treatment outcome of an intensive trauma-focused treatment program for post-traumatic stress disorder (PTSD) in older and younger adults. Methods: A non-randomized outcome study was conducted with 62 consecutively admitted older PTSD patients (60–78 years) and 62 younger PTSD patients (19–58 years), matched on gender and availability of follow-up data. Patients participated in an intensive eight-day trauma-focused treatment program consisting of eye movement desensitization and reprocessing (EMDR), prolonged exposure (PE), physical activity, and group psycho-education. PTSD symptom severity (Clinician-Administered PTSD Scale-5 (CAPS-5)) was assessed, at pre- and post-treatment, and for a subsample (*n* = 31 older; *n* = 31 younger patients) at six-month follow-up. Results: A repeated-measures ANCOVA (centered CAPS pre-treatment score as covariate) indicated a significant decrease in CAPS-5-scores from pre- to post-treatment for the total sample (partial *η*^2^ = 0.808). The treatment outcome was not significantly different across age groups (partial *η*^2^ = 0.002). There were no significant differences in treatment response across age groups for the follow-up subsample (pre- to post-treatment partial *η*^2^ < 0.001; post-treatment to follow-up partial *η*^2^ = 0.006), and the large decrease in CAPS-5 scores from pre- to post-treatment (partial *η*^2^ = 0.76) was maintained at follow-up (partial *η*^2^ = 0.003). Conclusion: The results suggest that intensive trauma-focused treatment is applicable for older adults with PTSD with a large within-effect size comparable to younger participants. Further research on age-related features is needed to examine whether these results can be replicated in the oldest-old (>80).

## 1. Introduction

In persons of 60 years and older, lifetime prevalence of post-traumatic stress disorder (PTSD) is about 4.5% [1] and has been found to be associated with considerable rates of comorbid psychiatric disorders, i.e., anxiety disorders (49.5%), mood disorders (47.1%), and personality disorders (32.5% [1]). A high disease burden of PTSD within older adults is apparent in them having very negative connotations concerning their quality of life [2] and with them experiencing more disability days than others without PTSD [3]. Furthermore, medical conditions affecting older adults are more common in patients with PTSD, such as dementia [4] and cardiovascular disease [5].

Despite the high disease burden, a systematic review [6] showed that there is a lack of well-designed intervention studies on PTSD treatment in older adults. Assessment, diagnosis, and treatment of PTSD in older adults has been shown to be challenging because of potential cognitive or sensory decline and comorbid physical disorders [7]. Another reason may be ageism, whereby therapists and older adults themselves have prejudices in thinking that treatment is no longer possible at old age [8]. These might be reasons that there are limited intervention studies available about older adults with PTSD [9]. Yet, general trauma-focused therapy has proven to be also effective in older patients [6]. Moderate to large effect sizes have been found in four randomized controlled trials (RCTs; [10,11,12,13] see Table 1). Compared to reported large effect sizes in a meta-analysis of randomized controlled trials for long-term efficacy of psychotherapy for PTSD in adults (Cohen’s *d* = 1.88 from pre- to post-treatment, and *d* = 2.14 from pre-treatment to follow-up [14]), results and effect sizes found in older adults are somewhat mixed and lower [6]. Reasons may be that older adults who experienced a traumatic event earlier in life may be more likely to have a chronic course of PTSD, possibly making symptoms less likely to change [6,10], especially over the long term [10]. Additionally, older adults are more at risk in experiencing worsening of PTSD symptoms during the course of treatment due to impairments in social support, financial stability, physical health, and cognitive functioning [6]. One published study compared Vietnam veterans (53–67 years, *M*age 59.43) and Operation Enduring Freedom and Operation Iraqi Freedom Veterans (20–50 years, *M*age 30.90) utilizing Cognitive Processing Therapy for PTSD [15]. They found higher post-treatment PTSD severity scores on the Clinician-Administered PTSD Scale (CAPS) for Vietnam veterans after controlling for pre-treatment CAPS score and number of sessions attended, which suggested that it may be more difficult to treat individuals who have had a chronic level of symptomatic disruption in their lives from PTSD and related symptoms. However, research specifically comparing the PTSD treatment effect between older and younger adults is missing.

In general, few studies have been carried out to compare the effects of psychotherapy in older and younger adults. Sabey et al. (2018) [16] concluded in their study in an outpatient mental health organization that older adults may change in similar fashion to middle-aged and younger adults in psychotherapy. Specifically for depression, differences in outcome between younger and older adults were not found [17] (patients). For anxiety disorders, mixed results were found whereby one meta-analysis showed moderate effect sizes for older adults compared to large effect sizes for middle-aged adults [18] (770 patients).

To maximize treatment outcomes, there has been increased attention to minimize time intervals between sessions [19]. In adults, there is evidence to suggest that if sessions are more frequently scheduled, more PTSD symptom improvement can be achieved compared to less intensive schedules [19].

In this study, prolonged exposure (PE) was combined with eye movement desensitization and reprocessing (EMDR) because distancing from the trauma and thus processing the trauma in a more “detached” manner may optimize treatment response [20]. Based on the fact that this may involve different working mechanisms, it is conceivable that both treatments could complement each other. Furthermore, adding physical activity could be promising way to improve treatment outcomes for people with PTSD [21]. Previous studies showed that an intensive program of eight days for PTSD, consisting of EMDR therapy, PE, physical activity, and group psycho-education, yields relatively high within treatment effect sizes in younger adults (Cohen’s *d* = 1.52−2.09 [22]; Cohen’s *d* = 1.87 [23]). Nevertheless, effect sizes of intensive trauma-focused treatment in older adults are missing.

We hypothesized that such an intensive program can also result in a large treatment effect in older adults (60+). Since treatment is given over a shorter time interval, the shorter time interval could serve as a proxy for a reduction of avoidance behavior, which is a PTSD-related symptom that seems to increase over the long-term course of PTSD in older adults [24] and could provide more support by more frequent therapist interaction. Furthermore, because previous meta-analysis did not find declined effect sizes for psychotherapy in older adults with depression [17], we specifically hypothesized that the decrease in PTSD symptoms in older adults is comparable to younger adults when applying an intensive trauma-focused treatment program.

## 2. Materials and Methods

### 2.1. Participants

Patients consecutively admitted between January 2018 and March 2019 (1443 patients) to the Psychotrauma Expertise Centre (PSYTREC), a Dutch mental health clinic specialized in the treatment of PTSD, were included as study participants. All patients were referred by a General Practitioner and had no history of previous treatment at PSYTREC. The referral criterion was a (probable) diagnosis of PTSD. Inclusion criteria for participation in the therapy program and research study were (1) being at least 18 years old, (2) a diagnosis of PTSD based on Clinician-Administered PTSD Scale (CAPS-5; [25]), (3) sufficient knowledge of the Dutch language to undergo treatment, and (4) not having a history of a suicide attempt in the three months prior to treatment.

**Figure 1:**
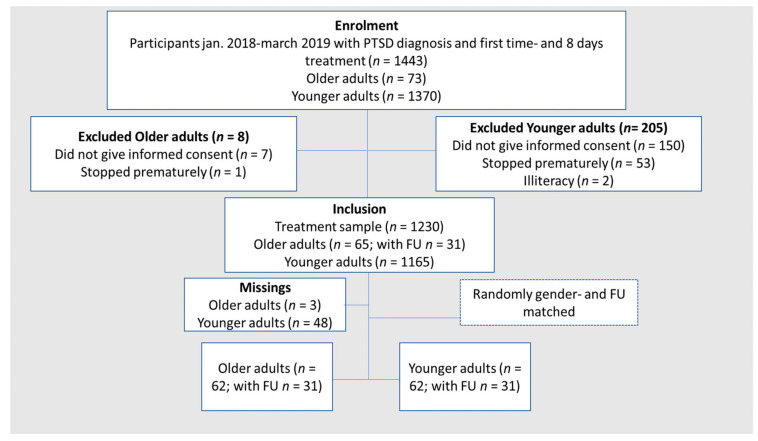
Flowchart of participants.

Of these patients, seven older and 150 younger adults did not give informed consent for research purposes. One older adult and 53 younger adults stopped treatment prematurely and two younger adults had illiteracy. The remaining 1230 participants fulfilled the full DSM-5 [26] diagnostic criteria for PTSD, which was established with the CAPS-5, but for three older adults and 48 younger adults, either the pre- or post-treatment measure was missing (Figure 1). For data analysis, 62 older adults were available, 31 including follow-up measurement. Based on the presence of follow-up data and gender matching (controlling for gender differences in PTSD [27]) with the older adult sample, we randomly selected 28 males (17 males with follow-up data, 11 males without follow-up data) and 37 females (14 females with follow-up data, 23 females without follow-up data) to obtain a matched sample of 62 younger adults (out of 1117 younger adults). We used the random sampling and allocation function in SPSS applied on the whole group younger male adults with follow-up (FU) and the whole group younger male adults without FU. Thereafter, we did the same for the younger female adults with and without follow-up data [28].

### 2.2. Procedure

All data were collected using the standard assessment instruments and regular monitoring outcome procedure of the PSYTREC mental health center. The study lacked random allocation: All older adults between January 2018 and March 2019 meeting inclusion criteria were included. All participants received the standard treatment procedure without adaptations. After the screening procedure, the patients received an intensive therapy program that took place within a time window of two weeks, consisting of 2 × 4 consecutive days of 8 sessions of PE (90 min per session) and 8 sessions EMDR therapy (90 min per session), alternated with periods of physical activities and group psycho-education sessions. After the first four days of treatment with overnight stay, patients went home. They returned for the final four days of treatment with overnight stay three days later. There was no preparation phase. Treatment was provided by the principle of “therapist rotation”, in which every patient was treated by multiple psychologists over the course of their treatment [29]. All psychologists were trained in PE and EMDR therapy administered according to standard treatment protocols [30,31]. The patients’ progress was discussed and monitored by a team of clinical psychologists and supervisors daily to secure treatment fidelity. Furthermore, fidelity and therapist adherence to the treatment protocol was verified during the supervision sessions. Nine days after treatment, patients returned to the center for the post-treatment CAPS-5 assessment. For a subsample, six-month follow-up data were available. Trained assessors administered the CAPS-5 at pre- and post-treatment and at six-month follow-up. They were blind to the study hypotheses. Ethical exemption of the study protocol was appointed by the Medical Ethics Review Committee of VU University Medical Center (registered with the US Office for Human Research Protections (OHRP) as IRB, FWA number FWA00017598).

### 2.3. Therapies

A slightly modified prolonged exposure protocol of Foa et al. (2007) [30] was applied in that sessions were not recorded, and no homework assignments were given. Furthermore, in vivo material and memorabilia of the processed traumatic events, such as pictures, clothes, or film parts were used in the PE sessions. Patients were asked to imagine the memories of the traumatic events as vividly as possible and had to describe their traumatic memories in the present tense, out loud and in detail. EMDR therapy was applied in accordance with the manualized standard EMDR protocol [31]. Trauma memories were recalled while at the same time eye movements were induced, to tax the patients’ working memory. Other additional possibilities to maximize working memory taxation were allowed including the use of a light bar, buzzers, or left/right clicking sound. Furthermore, standard cognitive interweaves were applied to facilitate processing [32], and the “flashforward protocol” [33] was employed in case of anticipatory fear and avoidance behavior. Alongside the individual PE and EMDR therapy, an intensive physical activity program in group format was offered, consisting of two timeslots of activities in the morning and two in the afternoon. All physical activity instructors were trained specifically for this program and had previous experience in working with PTSD patients. In the evenings, patients took part in psycho-education group sessions about, amongst others, trauma, PTSD symptoms, and the influence of physical activities.

### 2.4. Measures

To gather information about the potentially traumatic experiences, the Life Event Checklist for DSM-IV (LEC-IV) [25] was used from 1 January 2018 to 10 September 2018, and the Life Event Checklist for DSM-5 (LEC-5) [34] was used from 10 September 2018 to March 2019. Changes between LEC-IV and LEC-5 are minimal, and LEC-IV has a good temporal stability and reliability [35]. Given the minimal changes from the LEC-IV, few psychometric differences are expected for the LEC-5 compared to the LEC-IV. For older adults, normative studies are lacking. Scores were merged into main categories: (1) sexual abuse (sexual assault/unwanted sexual experience); (2) physical abuse (physical assault/assault with a weapon); (3) natural disaster, accidents and victims of war; (4) life-threatening illness, severe human suffering, and sudden death; (5) any other stressful event, captivity, and (satanic) torture.

To characterize the sample and to determine comorbid psychiatric disorders, the Mini International Neuropsychiatric Interview (MINI) [36,37,38] was administered. The MINI is a structured diagnostic interview developed on basis of the DSM-IV-TR criteria with good psychometric properties [38]. The kappa coefficient, sensitivity, and specificity have been found to be good or very good for most DSM diagnoses. Psychometric properties for older adults have not been investigated. In this study, MINI was used to focus on the presence of the most common comorbid mental diagnoses (depression, anxiety, and suicidal ideation).

Primary outcome measures were PTSD symptoms measured with the Clinician-Administered PTSD Scale-5 (CAPS-5) [39]. CAPS-5 is the gold standard to assess PTSD according to DSM-5 and it is a clinician-rated structured interview that was used to measure the symptom presence and severity of PTSD over the past month [27]. The interrater diagnostic agreement and internal consistency of the CAPS-5 and the Dutch version of CAPS-5 were excellent; the CAPS-5 total severity score showed high internal consistency (α = 0.90) and interrater reliability (ICC = 0.98, 95% CI: 0.94–0.99) [39]. Normative studies for older adults are not yet available for CAPS-5. It comprises the 20 DSM-5 PTSD symptoms clustered in four subscales (re-experiencing, avoidance, negative cognitions and mood, hyperarousal)—measured over the past month using 5-point scales for frequency (ranging from 0 = “never” to 4 = “almost daily”) and intensity (ranging from 0 = “none” to 4 = “extremely”). According to the basic CAPS-5 symptom scoring rule (SEV2 rule), a symptom is present if its severity is rated with 2 or higher [39]. By summing the symptom severity scores (criteria B-E), a total PTSD symptom severity score was computed. Higher scores indicated a higher PTSD symptom severity. CAPS-5 included two separate items representing the symptoms of the dissociative subtype: derealization and depersonalization. Using the score rule of frequency ≥2 and severity ≥2 on at least one of these items, patients were classified as meeting or not meeting the criteria of the dissociative subtype (as per [40]).

### 2.5. Data Analysis

Data were screened for outliers and missing data. No outliers could be identified, based on the interquartile range (IRQ; a data point is an outlier if it is more than 1.5 IQR above the third quartile or below the first quartile). If the CAPS-5 was administered, no items scores were missing. All analyses were performed with SPSS Statistics (version 25). Sample characteristics at baseline were described by means and standard deviations on the primary outcome measure (i.e., CAPS-5) and by the prevalence of clinical variables (i.e., MINI, LEC). Comparability of the groups at baseline was tested by *t*-tests (effect size Cohen’s *d*; CAPS-5) and chi-squared tests (effect size Cohen’s *h;* comorbidity rates, suicide risk, traumatic experiences). Rules of thumb for interpretation of the effect sizes [41] were for Cohen’s *d* and for Cohen’s *h* small 0.20, medium 0.50, and large >0.80.

Treatment outcome for the total sample (*n =* 124) and both younger age group (*n* = 62) and older age group (*n* = 62) was examined by comparing CAPS scores at pre- and post-treatment and for a subsample at six-month follow up of the younger age group (*n* = 31) and older age group (*n* = 31) using two general linear models (repeated measures ANCOVA) with age group (i.e., younger and older age group) as the between-subjects factor, and time (pre-treatment, post-treatment, and follow-up CAPS scores) as the within-subjects factor. To adjust statistically for pre-treatment differences between groups, we used the centered CAPS score at pre-treatment as a covariate. The covariate was centered by subtracting the mean covariates score from each covariate score, because in a classification design (i.e., a design including comparisons of participants samples from different populations, in this study younger and older age groups), a non-centered covariate measure would result in distorted estimation of within-subject factors, increase type I error, and lead to a loss of power [42]. Preliminary analyses indicated no assumptions were violated regarding normality, homogeneity of regression slopes, homogeneity of variance, and sphericity for the ANCOVA analysis. General rules of thumb for the interpretation of effect sizes were for *partial η*^2^, small = 0.01, medium = 0.13, and large ≥ 0.26 [43].

Furthermore, persistence of loss of PTSD diagnosis from post-treatment to follow-up for both the younger and older group was examined by a one-tailed *z*-score test (*p* < 0.05) to compare proportions.

## 3. Results

### 3.1. Baseline Sample Characteristics

The average age in the younger group (*n =* 62) was 39.5 years (SD = 11.32; range 19–58 years), and the average age in the older group (*n* = 62) was 64.1 years (SD = 4.36; range 60–78 years). Each group consisted of 54.8% women. Table 2 shows an overview of sample characteristics and differences between age groups at baseline. The two treatment groups reported severe PTSD symptoms as indicated by mean CAPS-5 scores. Furthermore, both groups had been exposed to a wide variety of multiple traumatic events, and there were high comorbidity rates (74.2%). In addition, 30.6% fulfilled the diagnostic criteria of the dissociative subtype of PTSD on CAPS-5, and a major group (41.9%) presented a moderate to high suicide risk (according to the MINI). A small proportion had missing data only for main diagnostic categories (anxiety and depression) on the MINI (*n* = 1 for depression; *n =* 3 for generalized anxiety). There were no significant differences between the two groups at baseline except for a medium effect in dissociative subtype, which was more frequently present in the younger group compared to the older group.

### 3.2. Treatment Effect from Pre- to Post-Treatment for the Total Group (n = 124)

**Figure 2:**
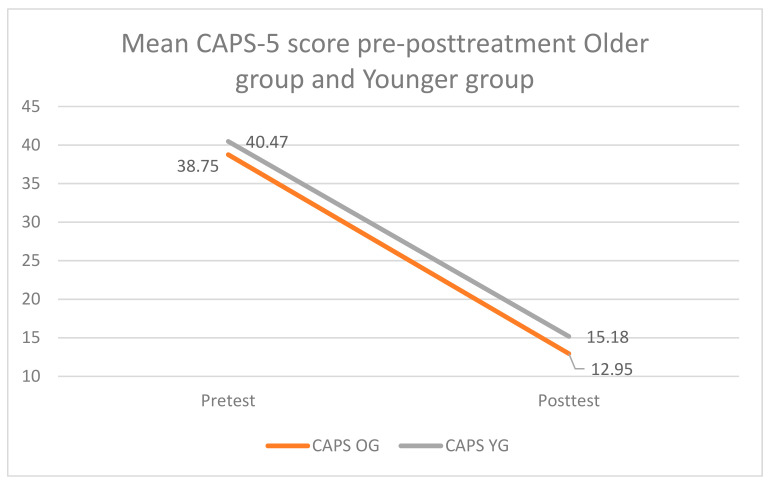
Pre and post-treatment mean scores for the older group and the younger group. Notes: CAPS = Clinician-Administered PTSD Scale; OG = older group; YG = younger group.

The mean pre- and post-treatment outcomes from the CAPS-5 for the younger (*n =* 62 and older (*n =* 62) age groups are displayed in Figure 2.

A repeated measures ANCOVA, with the centered CAPS-5 pre-treatment score as a covariate, indicated a significant and large decrease in CAPS-5 scores from pre- to post-treatment for the total sample (*n* = 124) (*F*(1, 121) = 509.705, *p* < 0.001, partial *η*^2^ = 0.808). The effect was not significantly different across age groups (*p* = 0.639, partial *η*^2^ = 0.002).

### 3.3. Treatment Effect at Six-Month Follow-Up for a Subsample (n = 62)

For the subsample (*n* = 62) with follow-up data available, the means and SDs from pre-treatment, post-treatment, and follow-up, and in younger (*n* = 31, *M* age = 40.3; SD = 10.28; range 21–58 years), and older (*n* = 31; *M* age = 65.1; SD = 4.65; range 60–74 years) age groups are displayed in Table 3.

A repeated measures ANCOVA, with the centered CAPS-5 pre-treatment score as a covariate, yielded a significant and large decrease in CAPS-5 scores from pre- to post-treatment (*F*(1, 59) = 184.776, *p* < 0.001, partial *η*^2^ = 0.76), and this treatment effect was maintained from post-treatment to 6 month follow-up (*F*(1, 59) = 0.192, *p* = 0.663, partial *η*^2^ = 0.003). There were no significant differences in treatment effects across age groups from pre- to post-treatment (*p* = 0.919, partial *η*^2^ < 0.001) and from post-treatment to follow-up (*p* = 0.538, partial *η*^2^ = 0.006). The majority of the follow-up subsample showed a loss of PTSD diagnosis at post-treatment (77.4% in the younger group (*n* = 24), 80.6% in the older group (*n* = 25)). These prevalence rates of loss of PTSD diagnoses were not significantly different across age groups (*p* = 0.76, Cohen’s *h* = 0.073). At 6 months follow-up, prevalence rates of loss of PTSD diagnoses were not significantly different across age groups (87.1% in the younger group (*n* = 27), 77.4% in the older group (*n* = 24); *p*= 0.32, Cohen’s *h* = 0.263). Furthermore, loss of diagnosis rates persisted at 6 months follow-up (*z* = −0.997, *p* = 0.16 for younger adults; *z* = −0.312, *p* = 0.38 for older adults).

## 4. Discussion

The results of the present study supported our hypothesis in that intensive trauma-focused treatment showed a large treatment response in both younger and older adults. Furthermore, there were no significant differences between the two age groups with respect to the decrease in PTSD symptom severity. In both age groups, within-treatment effects were large and were maintained at six-month follow-up.

The present study results, which included a higher frequency of sessions than usual, suggest that effects on PTSD symptoms are large in intensive formats, also in later life, compared to previous studies with medium effects on trauma-focused treatment in older adults with PTSD [6,11,13] with less inter-session intervals. A control condition with a lower frequency of sessions is needed to further explore the advantages of higher frequency of sessions. A study in primary care, which examined age differences in treatment response to a CBT collaborative care intervention (6–8 weekly sessions) for anxiety disorders (including PTSD) [44], suggested a more intensive treatment format and the use of booster sessions for older adults in order to increase treatment gains. To our knowledge, this is the first study that evaluated the feasibility of an intensive trauma-focused treatment for older adults with PTSD, and more importantly, it could demonstrate comparable treatment outcome for younger and older adults based on PTSD severity. These findings are on contrary to the findings of previous studies that have found mixed PTSD treatment effects in older adults [6] compared to younger adults [14], but they are analogous to psychological treatment results in depression [17] where no age differences were found. Furthermore, the combination with two different first-line treatments (EMDR and PE) and physical activity needs replication in control conditions with only EMDR or PE with and without physical activity, to evaluate the treatment effect of the different therapeutic elements on itself versus combining elements.

One of the major strengths of the present study is the inclusion of older as well as younger adults. Research on comparisons across age groups is a gap in treatment outcome studies for PTSD [6,10]. The current treatment sample contained not only younger adults but also a group of older adults (both male and female), with plenty of mixed and accumulated trauma. The participants in this study were gender-matched and shown to be a homogeneous group with no significant differences in pathology and trauma experiences between the age groups, with the exception of the dissociative subtype of PTSD, which was less prevalent in older adults, yet a similar outcome was also found in a previous study [45]. However, a recent meta-analysis found no evidence that dissociation symptoms moderate the effectiveness of psychotherapy for PTSD [46]. Another strength of the study was the use of common PTSD assessments and manualized, empirically-based treatment protocols with secured treatment fidelity, by supervision. Participants with concurrent mood, anxiety disorders, and suicidal ideation were not excluded, improving generalizability and clinical impact.

A first limitation of this study is the average age of the older adults, which was relatively low (64 years). More specifically, the age range from 60 up to 78 with mean age below 70 may make the findings less generalizable to the oldest-old (≥80 years). In general, the number of referred elderly participants was very low (*n* = 73 versus *n* = 1370 younger adults). This may indicate under-diagnosis and under-treatment due to ageism and negative stereotyping by referrers and older adults themselves, leading to lower use of mental health services [47]. Secondly, (age-related) comorbidities (e.g., somatic diseases, frailty e.g., healthcare dependency, functional decline, disabilities in daily life, cognitive symptoms) were not taken into account. Therefore, the impact of these comorbidities on the applicability and effect of intensive treatment in older adults remains an open question. Possibly, older, more vital, adults were referred to the intensive program, perhaps resulting in a selection bias, which might decrease the generalizability of the study. Older adults with more disabilities may need treatment with adaptations in an age-specific way [48]. Thirdly, although drop-out rates during the treatment program were low for both younger (3.9%) as well as older adults (1.2%), we still found high rates of attrition in our sample at follow-up (younger adults 60.3%; older adults 50%). We were unable to determine why individuals quit participating at follow-up and therefore, results at follow-up should be interpreted with caution. Fourthly, one of the methodological limitations is that the current study only used clinician-administered measures of PTSD symptomatology, and that CAPS-5 has as of yet not been validated for older adults. Finally, the lack of a control condition (e.g., waiting list) makes it difficult to rule out the possibility that the observed improvements during treatment were partly an artefact of time.

Clearly, more research is needed to replicate these findings in the oldest-old (≥80) and participants with sub-threshold PTSD. Additional measurements of age-related variables (somatic, cognition, self-efficacy) are required in order to gain understanding about possible factors that may affect treatment outcome and define (future) conditions in which a modified treatment approach is required.

In conclusion, these results suggest that intensive trauma-focused treatment is feasible and applicable for older, community dwelling adults suffering from severe PTSD, with large treatment response comparable to treatment response in younger participants.

## Figures and Tables

**Table 1:** Randomized controlled studies of trauma treatment studies including older adults.

Study	*n*	Mean Age	% Female	Trauma Type	Outcome Measure	Treatment Type	Number of Sessions	Outcome/Effect Sizes Cohen’s *d*
Lely et al. [11] (2019)	33	63.8	27.3	Mixed	CAPS	NET vs. PCT	11 sessions	Between group 0.44, disappears at follow up
Thorp et al. [10] (2019)	87	66.5	0	War	CAPS	PE vs. Relaxation	12 sessions	Within group 0.89, lost at 6 months follow up
Knaevelsrud et al. [13] (2017)	94	71.4	64.9	Childhood trauma	PDS	Therapist-Guided Internet-based Intervention vs. Waiting list	6 weeks intervention program, 11 sessions	Between group 0.42 maintained at 12 months follow-up
Bichescu et al. [12] (2007)	18	69.4	5.5	Political prisoner	CIDI	C1 NET vs. C2 psychoeducation	C1 5 sessions C2 1 session	Within group 3.15 (pre treatment-6 months follow up)

Notes: CAPS = Clinician-Administered PTSD Scale; NET = Narrative Exposure Therapy; PCT = Present Centered Therapy; PE = Prolonged Exposure; PDS = Post-traumatic Diagnostic Scale.

**Table 2:** Analyses of baseline characteristics of total, older, and younger treatment group with independent *t*-tests and chi-square tests.

	Total OG and YG	OG	YG	Significance Level	Effect Size
Mean CAPS score	39.61 (SD = 7.83)	38.75 (SD = 8.54)	40.47 (SD = 7.02)	*p* = 0.23	*d* = 0.22
Total comorbidity rates	74.2%	66.1%	82.3%	*p* = 0.12	*h* = 0.37
1 comorbidity	42.7%	37.1%	48.4%	*p* = 0.20	*h* = 0.22
2 or more comorbidities	31.5%	29%	33.9%	*p* = 0.56	*h* = 0.11
Moderate-high suicide risk	41.9%	38.7%	45.2%	*p* = 0.47	*h* = 0.12
Dissociative subtype	30.6%	21.0%	40.3%	*p* = 0.02	*h* = 0.42
More than 5 traumatic experiences	80.60%	82.30%	79.00%	*p* = 0.65	*h* = 0.08
More than 10 traumatic experiences	22.60%	22.60%	22.60%	*p >* 0.99	*h* = 0.00
Sexual abuse	83.10%	87.10%	79.00%	*p* = 0.23	*h* = 0.21
Physical	92.70%	90.30%	95.20%	*p* = 0.30	*h* = 0.19
Natural disasters, accidents, and victims of war	83.10%	85.50%	80.60%	*p* = 0.47	*h* = 0.06
Life-threatening illness, severe suffering, sudden death	89.50%	90.30%	88.70%	*p* = 0.77	*h* = 0.03
Any other stressful event, captivity, (satanic) torture	74.20%	74.20%	74.20%	*p >* 0.99	*h* = 0.00

Notes: CAPS = Clinician-Administered PTSD Scale; OG = older group; YG = younger group; SD = standard deviation.

**Table 3:** Pre, post-treatment and follow-up means and SDs, for the follow-up (FU) samples, older group (*n* = 31), younger group (*n* = 31), and total group (*n* = 62).

	Pre-test		Post-test		FU	
	Mean	SD	Mean	SD	Mean	SD
CAPS OG	40.06	9.88	15.90	14.26	16.10	15.28
CAPS YG	39.52	7.23	15.16	15.97	13.65	15.24
CAPS total group	39.79	8.59	15.53	15.02	14.87	15.19

Notes: CAPS = Clinician-Administered PTSD Scale; OG = older group; YG = younger group; FU = 6 months follow up; SD = standard deviation.

## Data Availability

The data presented in this study are available on request from the corresponding author. The data are not publicly available due to privacy.
